# Unusual extracellular appendages deployed by the model strain *Pseudomonas fluorescens* C7R12

**DOI:** 10.1371/journal.pone.0221025

**Published:** 2019-08-28

**Authors:** Dorian Bergeau, Sylvie Mazurier, Corinne Barbey, Annabelle Merieau, Andrea Chane, Didier Goux, Sophie Bernard, Azeddine Driouich, Philippe Lemanceau, Maïté Vicré, Xavier Latour

**Affiliations:** 1 Laboratoire de Microbiologie Signaux et Microenvironnement (LMSM EA 4312)—Normandie Université - LMSM, Evreux, France; 2 Agroécologie, AgroSup Dijon, INRA, Univ. Bourgogne, Univ. Bourgogne Franche-Comté, Dijon, France; 3 Structure Fédérative de Recherche Normandie Végétale 4277 (NORVEGE), Normandie, France; 4 Centre de Microscopie Appliquée à la biologie, SFR 4206 ICORE Université de Caen Normandie (CMAbio3), Caen, France; 5 Laboratoire de Glycobiologie et Matrice Extracellulaire Végétale—Normandie Université - EA 4358 Université de Rouen, Mont-Saint-Aignan, France; CINVESTAV-IPN, MEXICO

## Abstract

*Pseudomonas fluorescens* is considered to be a typical plant-associated saprophytic bacterium with no pathogenic potential. Indeed, some *P*. *fluorescens* strains are well-known rhizobacteria that promote plant growth by direct stimulation, by preventing the deleterious effects of pathogens, or both. *Pseudomonas fluorescens* C7R12 is a rhizosphere-competent strain that is effective as a biocontrol agent and promotes plant growth and arbuscular mycorrhization. This strain has been studied in detail, but no visual evidence has ever been obtained for extracellular structures potentially involved in its remarkable fitness and biocontrol performances. On transmission electron microscopy of negatively stained C7R12 cells, we observed the following appendages: multiple polar flagella, an inducible putative type three secretion system typical of phytopathogenic *Pseudomonas syringae* strains and densely bundled fimbria-like appendages forming a broad fractal-like dendritic network around single cells and microcolonies. The deployment of one or other of these elements on the bacterial surface depends on the composition and affinity for the water of the microenvironment. The existence, within this single strain, of machineries known to be involved in motility, chemotaxis, hypersensitive response, cellular adhesion and biofilm formation, may partly explain the strong interactions of strain C7R12 with plants and associated microflora in addition to the type three secretion system previously shown to be implied in mycorrhizae promotion.

## Introduction

Fluorescent pseudomonads are so-named because they produce soluble greenish pigments in conditions of iron limitation that fluoresce when illuminated with UV light [1]. These ubiquitous γ-proteobacteria have a considerable potential for adaptation to fluctuating environmental conditions, thanks to their highly versatile metabolism and the plasticity of their large genome [2–6]. This group of bacteria includes the species *Pseudomonas fluorescens*, which is generally considered to be a saprophytic rhizobacterium because its densities (10^6^ to 10^8^ CFU/g root) and metabolic activities are increased in the plant rhizosphere [7–10]. In addition, some strains protect plants from infection by interfering with phytopathogenic agents. This biocontrol activity generally involves competition with pathogens for space and/or nutrients, antagonism *via* the synthesis of toxic agents, antibiotics and biosurfactants, and stimulation of plant defenses [11–15]. The location and fitness of *P*. *fluorescens* render this antagonistic species even more powerful for treating diseases of roots and tubers, which are not generally accessible to germicidal treatments [16,17].

It is now widely accepted that the strong plant colonization and biocontrol capacities of fluorescent pseudomonads are associated with cell motility and chemotaxis towards the rhizosphere in addition to cell adhesion and biofilm formation on the roots [18–22]. These physical plant-bacterium interactions are closely associated with the presence of bacterial extracellular structures, such as flagella, fimbriae, pili and secretion systems [23–27]. Unfortunately, these nanostructures are only clearly visible on electron microscopy (EM), and they are therefore generally detected indirectly. Indeed, the presence of flagella, fimbriae and pili, which are involved in swimming, swarming and twitching motilities, respectively, is deduced from observations of bacterial growth on appropriate agar plates [24,28]. Likewise, evidence for the presence of a functional type three secretion system (T3SS) is provided by the observation of a hypersensitive response (HR) in tobacco leaves 24 to 48 hours after the infiltration of the bacterium concerned [29–31]. The difficulties encountered in observations of extracellular appendages result from their extremely small size (in the nanometer range) and brittleness: these delicate structures are generally destroyed by shaking during culture or during the transfer of cells onto the support for microscopic observation. Moreover, little is known about the environmental conditions triggering the synthesis of these components [32,33].

We performed ultrastructural observations by transmission electron microscopy (TEM). We observed several different membrane machineries on the rhizosphere-competent biocontrol strain *P*. *fluorescens* C7R12. This model strain harbors a remarkable set of extracellular structures, including a polar bundle of flagella, a putative T3SS that seems to be synthesized in response to fructose or trehalose induction, and bundles of dendritic fibrils forming a huge network of cells and microcolonies. We discuss the putative role of these structures in the primary steps of adhesion and biofilm formation.

## Materials and methods

### Bacterial strains and culture conditions

*Pseudomonas fluorescens* C7R12 [34] and a T3SS^*-*^ mutant derived from it, C7SM7, were used for these experiments. *Pseudomonas fluorescens* C7SM7 was obtained by site-directed mutagenesis of *hrc*C, a gene encoding an outer membrane pore forming protein required for type III-mediated secretion [35]. This mutant has been chosen since HrcC is required for the secretion of the HrpA protein forming the pilus of T3SSs belonging to the Hrp1 family [36]. Bacterial cells were cultured at 25°C in King’s B medium [37], a glycerol-rich medium with a low iron content that induces the synthesis of *Pseudomonas* siderophores and secondary metabolites [1,12], or in an *hrp*-inducing minimal medium (HIM) as described by Huynh et al. [38] and with the following composition: 1.7 mM NaCl, 1.7 mM MgCl_2_, 7.6 mM (NH_4_)_2_SO_4_, 50 mM KH_2_PO_4_, pH 5.7, supplemented with 10 mM glucose, fructose, sucrose or trehalose as the sole carbon source. For induction of the T3SS *hrp*A model gene, bacteria were first cultured in liquid KB medium to an OD at 600 nm of 0.6–0.8. The bacteria were washed twice in 10 mM MgCl_2_ and the OD_600_ was adjusted to 0.6 in liquid HIM. Induction was performed at 25°C, with shaking (180 rpm). KB medium was used to repress T3SS (i.e. as a non-inducing medium), to establish the basal level of *hrp*A gene expression (control).

### Swimming, swarming and twitching motility assays

Motility assays were performed on KB medium supplemented with 0.3%, 0.6% or 1% agar, for swimming, swarming and twitching motilities, respectively as previously described [39]. Briefly, swimming motility assay plates were inoculated with a toothpick. Swarming motility assay plates were inoculated by the deposition of 5 μl of overnight bacterial culture on the surface of the medium. Twitching motility assay plates were inoculated by depositing 5 μl of overnight bacterial culture underneath the medium, at the bottom of the Petri dish. The plates were incubated at 25°C for 48 h. Motility was assessed by measuring mean dendrite length for swarming, or the total diameter of the circular turbid zone for swimming. Twitching motility was assessed by determining the diameter of growth at the interface between the agar and the bottom of the Petri dish. This was achieved by removing the agar, washing the unattached cells of the plate with water, and staining the cells attached to the plate with crystal violet (1% [wt/vol] solution).

### Total RNA extraction and cDNA synthesis

Total RNA was extracted from 10^9^ bacteria by the hot acid-phenol method [40]. As required, a volume of culture containing 10^9^ bacteria was sampled and mixed with an equal volume of 100% ethanol 100% to stop transcription. Bacterial cultures were centrifuged at 4 000 × *g* for 10 min and the bacterial pellet was resuspended in 300 μl of lysis buffer (0.02 M sodium acetate, pH 5.5, 0.5% (w/v) SDS, 1 mM EDTA). Total RNA was then isolated by two rounds of extraction in 600 μl of hot acid phenol for three minutes each (pH 4.3; 60°C). The mixture was centrifuged at 13 000 × *g* for 15 min, and the aqueous phase was treated with 500 μl chloroform/isoamyl alcohol (24:1) and centrifuged as previously described. Total RNA was precipitated by overnight incubation of the supernatant at -20°C with two volumes of 100% ethanol containing 100 mM sodium acetate. The precipitated RNA was collected by centrifugation. The pellet was washed in 75% ethanol and resuspended in 50 μl DEPC water and treated with Ambion TURBO DNase (Life Technologies) for 2 h at 37°C. We checked for the absence of DNA contaminants by PCR with the standard NEB kit, using the 16S-FOR/16S-REV primers **(S1 Table)**. Total RNA was converted into cDNA by reverse transcription with the High-capacity cDNA RT Kit (Applied Biosystem).

### Quantitative RT-PCR

Specific primers for the target genes *hrp*A, and the 16S rRNA gene of *P*. *fluorescens* C7R12 were designed with Primer Express Software v3.0.1 **(S1 Table)**. Real-time quantitative amplification was performed with the 7500 Fast Real Time PCR system (Applied Biosystems). Reactions were performed in a 13 μl mixture containing 6.5 μl SYBR Green PCR Master Mix (2X SYBR Green 1 Dye, AmpliTaq Gold DNA Polymerase, dNTPs with dUTPs) with each primer present at a final concentration of 0.2 μM and 7.5 ng cDNA. The thermal cycling program was as follows: 95°C for 20 s, followed by 40 cycles of 95°C for 10 s, 60°C for 30 s and 72°C for 6 s, and then 95°C for 15 s, 60°C for 1 min and 95°C for 15 s. We used 16S rRNA as an internal control and the standard deviation in each case was below the 0.15 threshold cycle (CT). Relative quantification was performed as previously described, by the comparative CT (-2^ΔΔCT^) method [41].

### Preparation of samples for transmission electron microscopy

Bacteria were visualized on 200-mesh nickel EM grids (mesh diameter of 74 μm) coated with 2% Formvar (Leica Microsystems), a polyvinyl formal resin formed by polyvinyl alcohol and formaldehyde copolymerization with polyvinyl acetate, and produced by Monsanto Chemical Company (St. Louis, Missouri).

For the observation of flagella and the T3SS, assay conditions inducing the formation of these structures and preserving their integrity were established with a modified version of the protocol described by Roine et al. [42]. Bacteria were grown on solid KB or HMI medium supplemented with 10 mM fructose or trehalose as the sole carbon source, at 25°C for 48 h. The EM grids were then coated with the bacteria by transfer from the agar plate. The formvar-coated side of the grid was placed in contact with the bacterial lawn for 10 min to obtain a footprint of the cells and the organelles synthesized on the corresponding agar medium. For the observation of pili and dendritic fibril bundles, assays were performed according to an original protocol in which bacteria were cultured directly in liquid HIM on grids. The bacteria were first cultured overnight in KB medium. They were washed twice in 1 mM MgCl_2_, resuspended in HIM and the OD_600_ was adjusted to 10^8^ CFU/ml. A 20 μl droplet of this suspension was deposited in the center of a 9-cm Petri dish lined with wet filter paper. The EM grid was placed on the drop and the Petri dish was sealed with Parafilm. The plates containing the grids were incubated in a growth chamber at 25°C, in a saturated atmosphere, for 8, 12, 24 and 48 h.

Once the bacteria were loaded onto the grids, they were fixed by transferring the grids for 30 min onto a 20 μl drop containing 2% paraformaldehyde and 0.5% glutaraldehyde in PBS (pH 7.2, 137 mM NaCl, 2.7 mM KCl, 10 mM Na_2_HPO_4_ and 1.76 mM KH_2_PO_4_). Grids were then washed three times, for 5 minutes each, by placing them on a 50 μl drop of PBS and three times, for 5 minutes each, by placing them on a 50 μl drop of distilled water. The washed specimens were negatively stained by incubation for 10 s in 1% phosphotungstic acid, the pH of which was adjusted to pH 6.5 with KOH, and the specimens were then allowed to dry in air before viewing.

### Transmission electron microscopy

All observations were performed at PRIMACEN, the Normandy Cell Imaging Platform (Mont-Saint-Aignan, France), and/or at CMABio, the Center for Microscopy Applied to Biology (Caen, France) of Normandy University. Observations were performed on Tecnai 12 Bio-Twin (PRIMACEN) or JEO1 1011 (CMABio3) transmission electron microscopes operating at 80 kV. Images were acquired with an Erlangshen 500W (PRIMACEN) or Gatan Orius 200 (CMABio3) camera, and processed with Gatan Digital Micrograph software (on both microscopes).

### Statistical analyses

Cultures were set up in triplicate from three independent cultures, each of which was set up from an independent preculture. For quantitative PCR, we tested the null hypothesis that each expression value was not significantly different from the other expression values (*P* value calculated). *P* values below 0.05 were considered significant (*, *P* < 0.05). The standard deviation was below 0.15 *Ct* units. The results presented are the mean values from at least three independent experiments. Statistical analysis was performed with DataAssistTM software (v3.01), for the relative quantification of gene expression according to the comparative CT (2–ΔΔCT) method [41].

## Results

### Motilities and lophotrichous flagellation of *Pseudomonas fluorescens* C7R12

**Fig 1:**
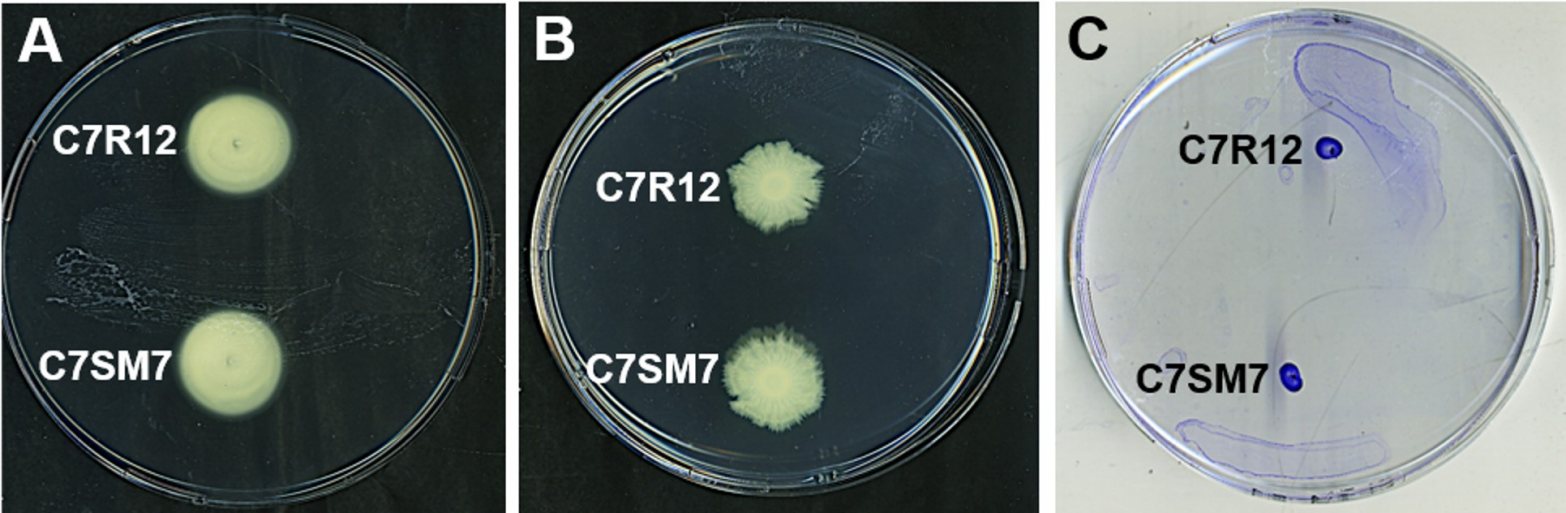
Swimming and swarming motilities of *P*. *fluorescens* C7R12 and a T3SS-negative mutant strain derived from it (C7SM7 strain). Cells were used to inoculate swimming (A), swarming (B) or twitching (C) motility assay agar plates containing King’s B medium, which were scanned after 48 h of incubation at 25°C. The results shown are representative of three independent experiments, each performed at least in triplicate.

Flagella are used in both swimming motility in liquids and swarming motility on wet semi-solid surfaces [43]. *Pseudomonas fluorescens* C7R12 strain and the T3SS-defective mutant derived from it, C7SM7, both displayed swimming **(Fig 1A)** and swarming **(Fig 1B)** motilities in KB agar. For swimming, the mean of the diameter of the turbid zone was similar for the two strains (27.4 ± 1.3 mm for the C7R12 strain and 28 ± 1.3 mm for the C7SM7 strain). In the most studies of *Pseudomonas aeruginosa*, a flagellum with a polar insertion is responsible for this mode of motility and is generally observed in aqueous environments [44]. Unlike swimming, swarming motility requires a concerted multicellular effort, biosurfactant secretion to decrease the surface tension of the swarm fluid and, in some cases, an increase in the number of flagella [45]. Swarming appears to be a mode of motility enabling bacterial populations to colonize surfaces rapidly, resulting in biofilm formation [43]. The C7R12 and C7SM7 strains were able to migrate away from the initial location collectively, with a mean growth diameter of 32.2 ± 2 mm measured for C7R12, and of 31.9 ± 3.1 mm for C7SM7, in our KB agar conditions. The swarming observed took the form of small fringes **(Fig 1B)** as previously described for other *P*. *fluorescens* strains [46]. However, this swarming did not result in the formation of the expanding irregular branching pattern typical of swarming motility in *P*. *aeruginosa*, as described by Rashid and Kornberg [47].

Twitching motility is a mode of translocation over semi-solid or solid surfaces in humid conditions. It is dependent on the presence of retractile type IV pili, but does not require flagella [48]. In twitching motility, translocation across the surface is mediated by the extension and retraction of pili and corresponds to a collective behavior supporting colony expansion [48]. In our twitching motility assays, both strains formed a dense crystal violet-stained spot, the diameter of which corresponded to that of the initial inoculum (5 μl) **(Fig 1C)**. Thus, these strains were unable to move over the surface of the Petri dish, but they were able to adhere to it. We can therefore conclude that these *P*. *fluorescens* strains have no twitching motility and probably do not produce type IV pili under our experimental conditions.

**Fig 2:**
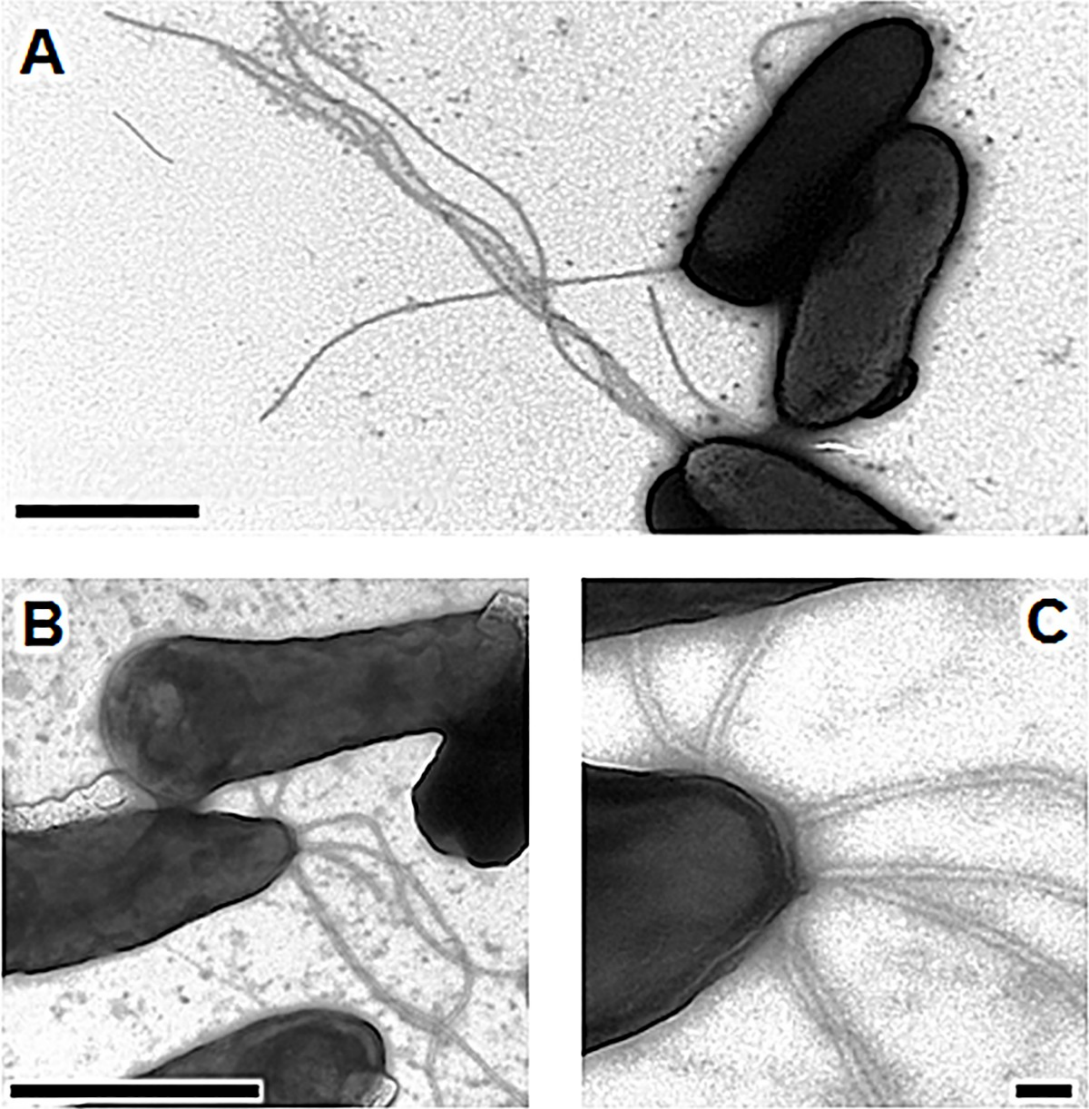
Production of flagella by *P*. *fluorescens* C7R12. Cells were grown for 48 h at 25°C on plates containing solidified King’s B medium. They were then transferred by footprint to the electron microscopy grids and negatively stained with phosphotungstic acid. The cells harbored a lophotrichous cilium, generally composed of multiple long flagella (A). Up to seven flagella were observed emerging from a single bacterium, as shown in (B). An enlargement of this image reveals polar insertion details and can be used to estimate the diameter of each pilus (C); the sizes of the scale bars are 1 μm (A,B) and 0.1 μm (C).

We investigated the type of cilia responsible for the swimming and swarming of the C7R12 strain, by culturing this strain on KB agar plates and then transferring it to an EM grid. Cells were negatively stained and examined by high-resolution TEM. *P*. *fluorescens* C7R12 was rod-shaped, about 2 μm long and usually covered with four to seven flagella all connected to the cell body at the same polar position **(Fig 2A and 2B)**. In our assay conditions, these flagella were about 4 to 5 μm long and had a diameter of about 20 nm. In some bacteria, the base of the multiple flagella is surrounded by a specialized region of the cell membrane known as the polar organelle [49]. No such polar organelle was observed here **(Fig 2C)**. The multiple flagella are thought to act in concert to drive bacterial swimming by pushing, pulling or coiling around the cell body in the vicinity of a semi-solid agar surface [50], consistent with our findings **(Fig 1)**.

### The expression of *hrp*A, encoding the main protein of the T3SS pilus, is strongly induced by fructose, sucrose and trehalose in minimal medium

The T3SS apparatus is not constitutively produced by bacteria and remains difficult to observe without specific and favorable assay conditions [33,51]. Various culture media favoring the production of T3SS in the phytopathogen *Pseudomonas syringae* have been described. We chose to use a basal salt medium mimicking the composition and pH of the plant apoplast [38,52]. These conditions are thought to trigger the production, by bacteria, of a T3SS injectisome close to the potential target (i.e. plant cells) [32,38,53]. We added various plant sugars to this minimal medium, and compared their ability to act as a carbon source and to induce the production of the T3SS by the *P*. *fluorescens* C7R12 strain.

**Fig 3:**
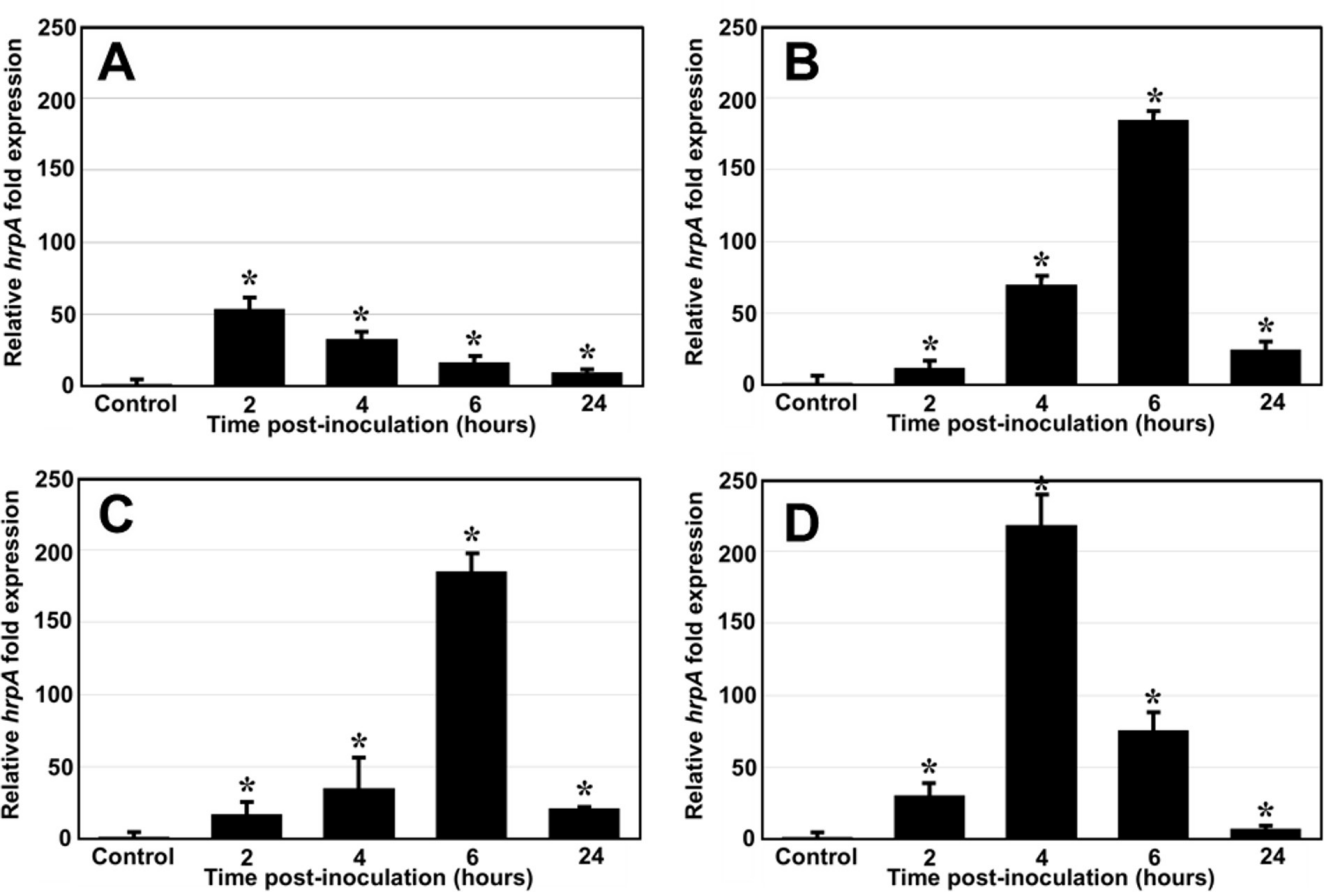
Induction of *hrpA* expression in *P*. *fluorescens* C7R12 cultured in HIM supplemented with mono- or disaccharides. We analyzed *hrp*A transcription by RT-qPCR on the *P*. *fluorescens* C7R12 strain grown at 25°C in *hrp*-inducting minimal medium supplemented with 10 mM glucose (A), fructose (B), sucrose (C) or trehalose (D). Levels of *hrp*A expression are expressed relative to those obtained on King B (non-inducing) rich medium. The data shown are the mean values obtained in three independent experiments. Statistical analysis was performed with DataAssistTM software (v3.01), with relative gene expression levels quantified by the comparative CT (2^–ΔΔCT^) method. The significances of differences between mean values were determined by calculating *p-*values in Student’s *t* tests (*P < 0.05).

We assessed the efficacy of this medium for inducing T3SS by first evaluating the relative expression level of a key T3SS gene, *hrp*A, which is known to be highly expressed and representative of HrpL-regulated T3SS genes in *P*. *syringae* [32,33]. This gene encodes the major structural protein of the T3SS pilus, enabling the pilus to elongate until it reaches its target, through polymerization [54]. RT-qPCR analysis was performed to compare *hrp*A expression between the C7R12 strain cultured in liquid HIM medium and in KB medium. With glucose as the inducer, *hrp*A transcription levels in HIM were significantly higher than those observed in KB cultures, with a maximum 50-fold difference after 2 h of incubation **(Fig 3A)**. Higher fold-changes were obtained for media containing fructose, sucrose or trehalose in place of glucose. Fructose and sucrose induced a maximum fold-change of about 180 after 6 h of incubation **(Fig 3B and 3C)**. The similarity of the transcription levels obtained with these two sugars may be explained by the composition of sucrose, a disaccharide with fructose as one of its components. Interestingly, stronger *hrp*A induction (around 220-fold) occurred when the C7R12 strain was cultured in HIM supplemented with trehalose **(Fig 3D)**. We therefore chose to use HIM supplemented with fructose or trehalose for subsequent TEM studies.

### Occurrence and architecture of type III secretion systems

The induction of HR, a form of localized programmed plant cell death at the site of infection, suggests that effector proteins are translocated into the plant cell via a T3SS [29–31]. A HR can be generated only if the bacterium harbors an operational T3SS and injects effector proteins into the plant cell [55–57]. *P*. *fluorescens* is considered to be a “safe” saprophytic rhizobacterial species with no phytopathogenic potential [3,4]. It does not, therefore usually elicit a HR [58]. C7R12 differs from most *P*. *fluorescens* strains in its ability to elicit a HR similar to that recorded with *P*. *syringae* pv. tomato DC3000 on tobacco leaves within 24 hours, and the presence of T3SS gene sequences very similar to those present in this reference strain [35,59]. By contrast, the T3SS mutant C7SM7 was unable to so induce a HR, indicating that the HrcC structural protein targeted during the construction of this mutant is an indispensable element of the architecture of the T3SS [35]. We therefore used the C7SM7 strain as a negative control with no T3SS appendage.

**Fig 4:**
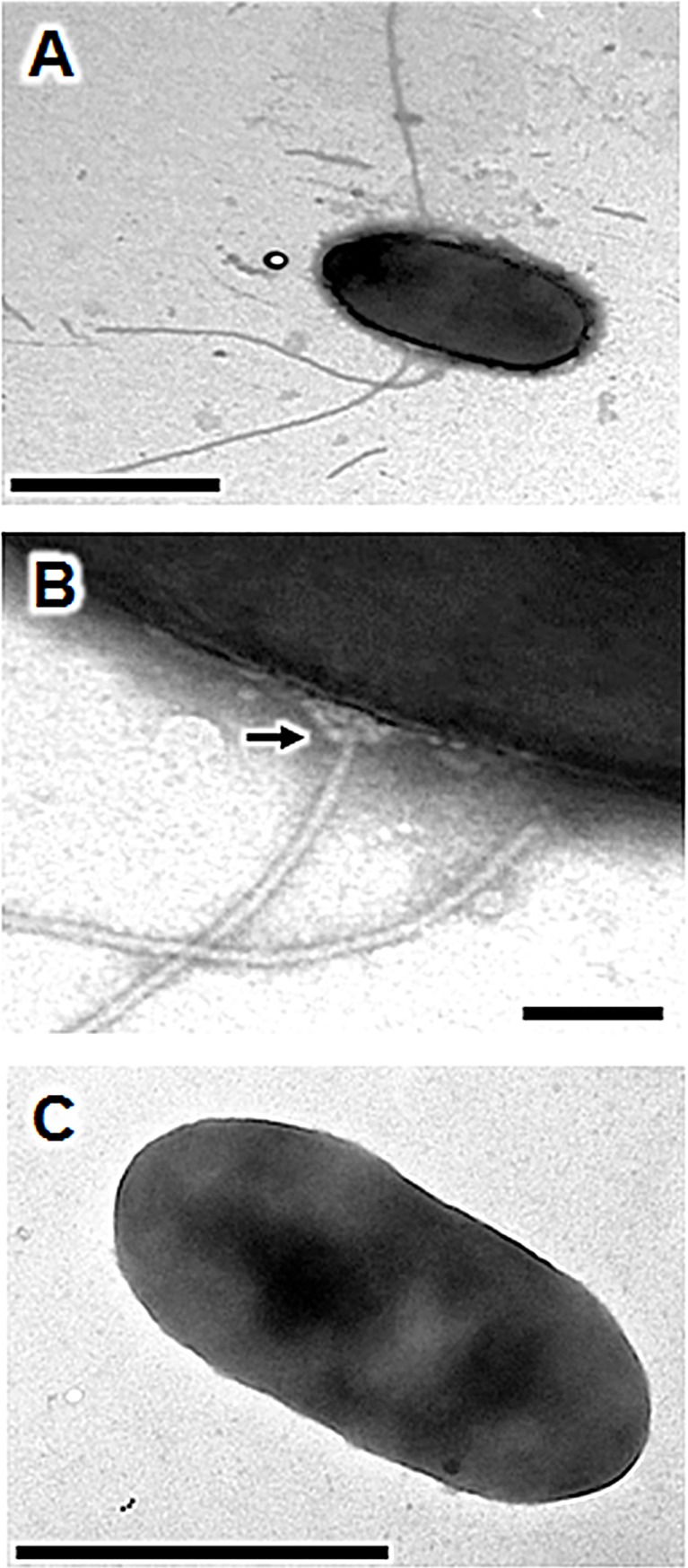
Production of a type three secretion system (T3SS) by *P*. *fluorescens* C7R12. Cells were grown for 48 h at 25°C on solidified *hrp*-inducing minimal medium supplemented with fructose (10 mM). They were then transferred by footprint to the electron microscopy grids. Transmission electron micrographs of negatively stained *P*. *fluorescens* C7R12 cells showing (A) three non-polar long and thin putative Hrp pili. (B) A magnification of the image in A shows the presumed T3SS basal body (black arrow) embedded in the outer membrane of this Gram-negative bacterium; (C) Under these assay conditions, the T3SS-negative mutant C7SM7, used as negative control, is deprived of appendages; the sizes of scale bars are 1 μm (A,C) and 0.1 μm (B).

The T3SS manufactured by *P*. *syringae* phytopathogens belong to the Hrp1 family [60]. They form long thin filaments that may extend from 200 nm to several micrometers in length, with a diameter of 10–12 nm, in laboratory conditions [54]. The TEM micrographs obtained in our assay conditions revealed putative T3SS appendages of about 700 to 1,750 nm in length, with a diameter of about 10 nm. Unlike the polar flagella, the T3SS pili were always positioned on the elongated part of the cell body **(Fig 4A)**. In this context, we also observed the basal portion of the pilus inserted into the outer membrane, which formed a ring (presumed to be the HrcC hexamer) from which the pilus extended **(Fig 4B),** as already reported for *P*. *syringae* DC3000 [53]. As expected, we observed no such structures in the C7SM7 *hrc*C^-^ mutant (**Fig 4C**). Thus, *P*. *fluorescens* C7R12 produces an extracellular apparatus similar to those of Hrp1-T3SS family, as visualized in *P*. *syringae* DC3000 [42,53,61]. This finding is entirely consistent with the previous Hrp1-like classification of the *hrc/hrp* gene cluster in the C7R12 genome [59,62].

### Deployment of a network of dendritic fibril bundles

**Fig 5:**
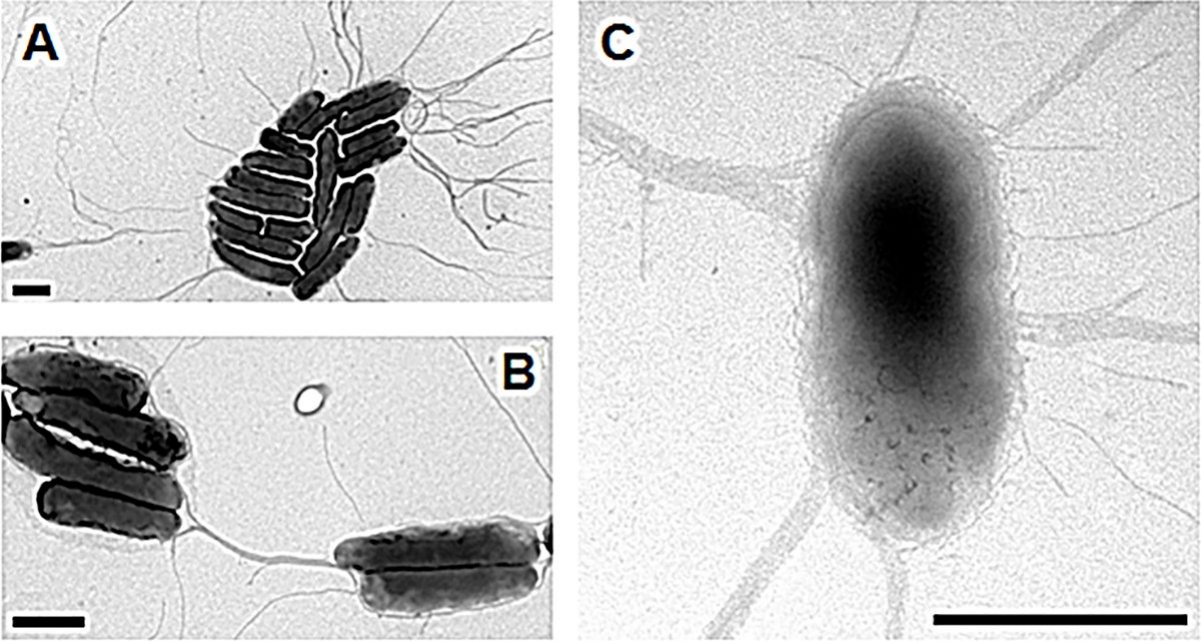
Production of pili and fibrils by *P*. *fluorescens* C7R12 and its T3SS-negative mutant C7SM7. Cells were grown at 25°C on formvar electron microscopy grids on a drop of *hrp*-inducing minimal medium supplemented with fructose or trehalose (10 mM). Transmission electron micrographs of negatively stained *P*. *fluorescens* C7R12 (A) and its *hrc*C^-^ mutant C7SM7 (B) microcolonies or single cells (C). *P*. *fluorescens* C7R12 and the T3SS mutant C7SM7 produced similar fibrils from 8 h of incubation onwards. These fibrils encircled both single cells (C) and microcolonies (A,B) and connected them (B); the size of the scale bars is 1 μm.

Most TEM specimens must be supported on a thin electron-transparent film, to hold the sample in place. Formvar films are thermoplastic resins composed of polyvinyl formals. They are a frequent choice of film grid for TEM because they allow the use of grids with a lower mesh rating (see Materials and Methods section). When the C7R12 and C7SM7 strains were cultured directly in a drop of HIM placed on the surface of an abiotic formvar grid, the cells rapidly deployed novel extracellular structures. We observed long, thick pili associated to various extents into fibrils, which eventually formed fibril bundles, depending on growth stage. After 8 hours of culture, fibrils seemed to be synthesized by microcolonies consisting of a few individual cells to several dozen cells. These fibrils seemed to pack the bacterial wall or the border of the whole colony, forming a fibrous capsule connecting individuals or microcolonies to each other **(Fig 5A and 5B)**. In rare cases, the fibrils appeared to be produced by and collected around a single cell. However, we never clearly observed a pilus or fibril anchored in the cell membrane or localized to a particular part of the cell, such as the pole, and we were unable to detect potential sites of insertion into the cell **(Fig 5C)**. One possible reason for this is that *P*. *fluorescens* cells release their pili proteins or components into the extracellular compartment in order for binding to the fibrils to lengthen them.

**Fig 6:**
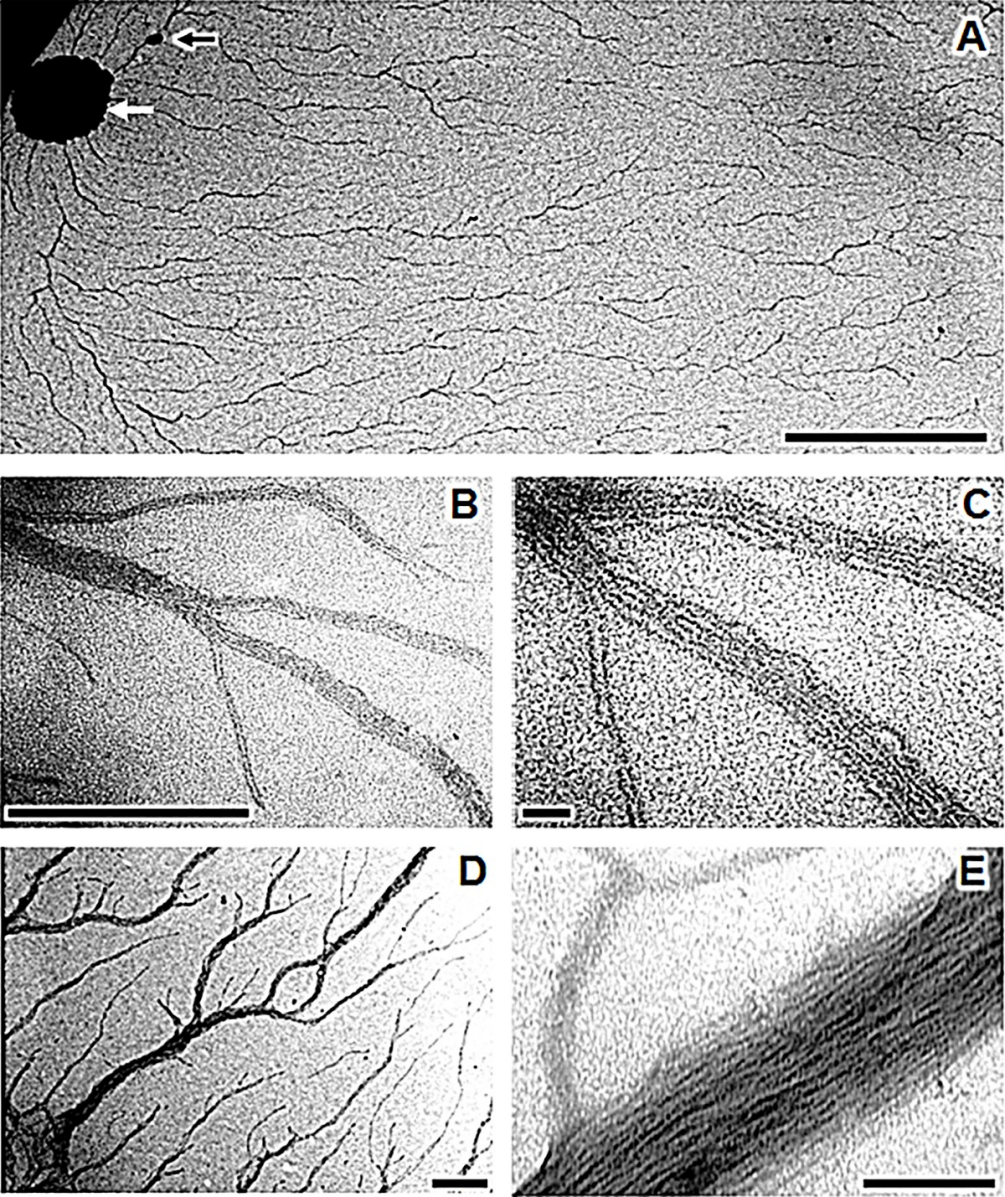
Fibril bundle network deployed by *P*. *fluorescens* C7R12. Cells were grown at 25°C on formvar electron microscopy grids on a drop of *hrp*-inductive minimal medium supplemented with fructose (10 mM). (A) Extended dendritic network of fibrils produced by a microcolony of about a hundred cells (black arrow) and a single cell (white arrow) after 24 h. (B) Apex of a fibril with its extension fork. (C) A magnification of the image in B showing the detailed structure of fibrils composed of one to five contiguous pili. (D) Dense fibril bundles obtained after 48 h. (E) A magnification of the image in D showing the ramification of a bundle composed of several dozen fibrils; the sizes of the scale bars are 10 μm (A), 1 μm (B,D), and 0.1 μm (C,E).

After 24 hours, microcolonies including a few hundred individuals were able to deploy a fractal network of fibril bundles over an area of up to about a 1000 μm^2^ **(Fig 6A)**. The bundled fibrils seemed to be closely associated with microcolony structures. Each fibril consisted of large parallel arrays of two to eight thin packed individual pili with diameters of about 10–15 nm. The extremities of each bundle displayed branched dendritic growth and subdivision, optimizing the exploration and colonization of the contact surface **(Fig 6A and 6B)**. At this stage, individual pili were rarely observed in the fibrils, except at the growing extremities of the bundle **(Fig 6B and 6C)**. After 48 hours, the bundles of fibrils appeared darker on TEM, as they had become larger and denser **(Fig 6D)**. An analysis of high-magnification images of a single fibril revealed a regular structure resembling the stacking of several dozens of pilus subunits **(Fig 6E)**.

## Discussion

*Pseudomonas fluorescens* C7R12 is a strain isolated from a soil naturally suppressive to Fusarium wilt. It has been shown to be an effective biocontrol agent [62,63], to protect *Medicago truncatula* against *Pythium* [35], to promote plant growth and arbuscular mycorrhization [35,64,65], and to colonize the rhizosphere and the root tissues of various plant species efficiently [34,64,66]. These abilities have been shown to be associated with (*i*) the presence of a functional primary metabolism coupling aerobic and nitrogen oxide respiration [67,68], (*ii*) an ability to scavenge efficiently from the environment [34], (*iii*) a capacity to synthesize a siderophore (pyoverdine) with a very high affinity for iron and an ability to improve plant iron nutrition [69–71], (*iv*) the presence of a functional T3SS [35], and (*v*) an ability to assimilate specific compounds from fungi and plants (e.g. sucrose, trehalose) [9,72]. By contrast, the ability of *P*. *fluorescens* C7R12 to move, adhere to the root surface, or develop physical interactions with root cells or microbial partners (e.g. mycorrhizae) in the rhizosphere has yet to be evaluated, and the existence of extracellular appendages potentially involved in these behaviors has not been investigated.

The bacterial flagellum is an apparatus composed of more than 20 different proteins, with a basal body that crosses the cell wall and is connected to the flagellar filament by a hook, serving as a flexible joint to change the angle of flagellar rotation [25,73]. It is an effective locomotion organelle that enables bacteria to achieve speeds exceeding many cell body lengths per second [74]. Flagella have also been reported to function as adhesins, playing a key role in bacterial adhesion and virulence [25]. Members of the species *P*. *fluorescens* have traditionally been considered to have a single flagellum at one of the cell poles, the so-called “monotrichous” conformation [22,75,76]. However, cells of *P*. *fluorescens* C7R12 have an unusual lophotrichous ciliature, with a polar tuft of four to seven flagella. Bacterial motility plays a key role in dispersion and surface colonization in soil-resident bacteria, such as *P*. *fluorescens* [19,77]. This motility is dependent on the number of flagella and their arrangement on the cell body [78]. Hintsche et al. [50] showed that a *Pseudomonas putida* strain with a polar tuft of helical flagella could use different swimming patterns, with the bacteria able to move as “pushers” or “pullers” or to propel themselves with the bundle of flagella wrapped around the cell body. The strong rhizosphere-competence of the C7R12 strain may, thus, be at least partly due to the presence of this atypical multiple flagellation, increasing the speed of movement (hypermotility), and altering the nature of displacements **(Fig 1)**, thereby increasing fitness and chemotaxis capacity in the vicinity of plants [75,79]. However, unanswered questions remain about the role of this unusual flagellation in the adhesion process.

Type three secretion systems have been shown to play a determining role in host interactions mediated by fluorescent pseudomonads, including the opportunistic pathogen of animals and humans *Pseudomonas aeruginosa* and the plant pathogen *Pseudomonas syringae* [80,81]. In these pathogenic species of *Pseudomonas*, the T3SS is involved in cell-to-cell contact with the eukaryotic host and in bacterial virulence. Genes encoding the basic structural elements of the T3SS are conserved among Gram-negative bacteria [60,82]. Eight families of T3SSs have been described on the basis of sequence analyses [83,84]. The T3SS of *P*. *aeruginosa* involved in bacterial virulence belongs to the Ysc-T3SS family. It forms a short needle-like structure that acts as an injectisome, delivering toxins to the target cell cytosol [85,86]. The T3SS of *P*. *syringae*, which has been implicated in plant pathogenicity, belongs to the Hrp1-T3SS family. It forms a long, thin, flexible pilus, capable of penetrating the thick walls of plant cells. This pilus is essential for the injection of multiple effector proteins into plant cells to suppress plant innate immune defenses, manipulate hormone signaling and trigger cell death [30,57,80,87]. *P*. *fluorescens* is considered to be non-phytopathogenic, but the presence of T3SS genes related to those of the Hrp1-T3SS family has been reported in some rhizosphere isolates [83,88–94]. However, only a minority of these strains can trigger a HR, suggesting that the *hrc/hrp* gene cluster present is generally incomplete and insufficient for the production of an operational T3SS machinery [58]. Thus, little is known about the architecture of the T3SS carried by *P*. *fluorescens* strains other than *P*. *fluorescens* 2P24 [90]. This strain has a filament of more than 1 μm in length, with a diameter of about 13 nm, at the end of there is a basal body composed of two rings, each about 20 nm across. Unfortunately, in one study in which the T3SS machinery was extracted from *P*. *fluorescens* 2P24 cells by CsCl gradient centrifugation, it was not possible to determine the site of insertion of the T3SS into the cell body [90]. The existence of a functional T3SS in strain C7R12 has been demonstrated, but this machinery was not observed directly [35]. In our assay conditions, TEM provided direct visual evidence of the existence in this strain of a complete T3SS machinery similar to that of Hrp1-T3SS from *P*. *syringae* and *P*. *fluorescens* 2P24 [42,53,61,90]. This assertion is based on the typical architecture, size and localization of the putative T3SS detected and because this appendage is observed in the C7R12 strain but not in the C7SM7 mutant, under the same assay conditions (**Fig 4**). It would be interesting in a further study to work on the labeling of proteins characteristic of the extracellular part of T3SS such as HrcC, HrpA or HrpZ to reinforce the characterization of this appendage and better study its role in the interactions between the bacterium and its environment.

The putative T3SS of *P*. *fluorescens* C7R12 was observed only during culture in an *hrp*-inducting minimal medium in the presence of fructose, which has been reported to be necessary for the observation of the T3SS in *P*. *syringae* strains [42,53,61], or in the presence of trehalose. We first checked the expression of the *hrp*A gene, as a master controller of T3SS gene expression, in HIM supplemented with mono- (glucose, fructose) or disaccharides (sucrose, trehalose). Interestingly, trehalose was the strongest and earliest inducer of *hrp*A gene expression **(Fig 3),** a finding never before reported, to our knowledge. This compound is a nonreducing sugar found in many organisms, including fungi and plants. In plants, the activated form of the molecule (trehalose-6-phosphate) is a key signal molecule that regulates carbon assimilation and sugar status [95–97]. For fungi, including plant-associated mycorrhizae, trehalose is both a high-energy compound and can be used for glucose storage and osmotic regulation, these tradeoffs favoring its preferential use. It can account for as much as 20% of fungal biomass [98–100]. The presence of trehalose in the environment may alert strain C7R12 cells to the presence of target host-plant or fungal cell, triggering the production of T3SS. This is consistent with (*i*) the involvement of the T3SS in promoting arbuscular mycorrhization by C7R12 [35], and (*ii*) more generally, the assimilation of trehalose as a substrate by fluorescent pseudomonad populations associated with the rhizosphere and mycorrhizosphere, but not by pseudomonad populations isolated from bare soil [9,101,102]. It has been previously shown that populations of fluorescent pseudomonads harboring T3SS genes were more abundant in mycorrhizospheres than in other environments [27, 59, 62]. Moreover, it has been demonstrated that the T3SS of the strain C7R12 was responsible for the promotion of mycorrhization of *Medicago truncatula* because root colonization by arbuscular mycorrhizal fungi was not promoted by a T3SS-negative mutant [35]. These findings are in agreement with our results.

When *P*. *fluorescens* C7R12 or C7SM7 was cultured on EM grids containing HIM, the bacteria adhering to the formvar surface formed microcolonies characterized by long, filamentous fibrils composed of bundles of individual pilus strands. These fibrils appeared to be synthesized in a disordered manner close to the bacterium or microcolony **(Fig 5),** subsequently becoming organized and joining together to form long branched bundles up to 50 times the size of the bacteria in our test conditions **(Fig 6)**. These structures strikingly resembled the bundles of fimbrial low-molecular-weight protein (Flp) pili first observed by Kachlany et al. [103,104] in *Aggregatibacter* (formerly *Actinobacillus*) *actinomycetemcomitans*, the causal agent of aggressive periodontitis. The other available data suggest that these proteinaceous appendages are related to structures referred to by different authors as the “tight adherence pilus” (Tad) or “rough colony protein” (Rcp) in addition to Flp [105]. The *tad* genes encode the machinery required for the assembly of adhesive Flp pili, including their major structural glycoprotein component, which is encoded by the *flp-1* gene [106]. They are essential for biofilm formation, colonization and pathogenesis in the genera *Aggregatibacter*, *Haemophilus*, *Pasteurella*, *Yersinia*, *Caulobacter* and among the fluorescent pseudomonads of the species *Pseudomonas aeruginosa* (for review see Tomich et al. [107]). Interestingly, *tad* genes have also be implicated in the plant pathogenicity of two potato pathogens, *Pectobacterium* and *Ralstonia* [108,109]. However, among published TEM studies of Flp structures [109–111], images only of Flp fibrils from *A*. *actinomycetemcomitans* [104,106], and, to a lesser extent, *Ralstonia solanacearum* [112], revealing the structure and size of these fibrils to be similar to those recorded for *P*. *fluorescens* C7R12 and C7SM7 **(Fig 6)**. Bhattacharjee et al. [113], concluded that Flp fibrils were not involved in mobility because they are devoid of the PilT ATPase essential for pilus retraction phenomena, as observed in twitching motility [114]. This is probably also the case for the C7R12 and C7SM7 strains, which displayed no motility in twitching conditions, including culture in HIM, but, conversely, displayed an adhesion of colonies to the surface of Petri dishes **(Fig 1)**. By contrast, Flp fibrils contribute to the non-specific adhesion of bacteria to abiotic surfaces (glass, stainless steel) or eukaryotic (mammalian) cells, and they promote bacterial aggregation for the formation of microcolonies and virulence [104–106]. *P*. *fluorescens* pili clearly act not only in parallel arrays to form thick fibrils, but also in adhesion to environmental surfaces **(Fig 6)**. Precisely, the synthesis and assembly of both flagella and Flp pili have recently been shown to be key determinants of plant root colonization [23].

In conclusion, our study provides direct evidence for the existence, in *P*. *fluorescens* C7R12, of an extracellular apparatus unusual in non-pathogenic fluorescent pseudomonads. This apparatus includes a bundle of polar flagella, thin flexible pili resembling Hrp1-T3SS injectisomes and densely bundled fimbria-like appendages protruding from the entire surface of the microcolony to form a wide fractal network, strongly suspected to support preliminary steps in *Pseudomonas* biofilm deployment. The nature and anchoring of these structures depend on the composition (e.g. sugars), hydrophobicity and density of the cellular microenvironment. Further studies are required to determine the precise role of these various appendages and to check for their existence in other rhizosphere-competent and biocontrol strains.

## Supporting information

S1 Table Primers used for RT-qPCR assays. Underlined sequence of primers used both for 16SrRNA and *hrp*A RT-qPCR assays.
(PDF)
