# Supplementary material for: Unusual extracellular appendages deployed by the model strain Pseudomonas fluorescens C7R12
Source: PLoS One. 2019 Aug 28;14(8):e0221025. doi: 10.1371/journal.pone.0221025 (PMC6713353; doi:10.1371/journal.pone.0221025)
Supplement: S1 Table — Underlined sequence of primers used both for 16SrRNA and hrpA RT-qPCR assays. (PDF) [file pone.0221025.s001.pdf]

Supporting information

Unusual extracellular appendages deployed by the model strain *Pseudomonas fluorescens* C7R12

by Bergeau et al. (2019)

S1 TABLE. Primers used for RT-qPCR assays

Primer name	Sequence*	Reference
**16SrRNA**		
16S-C7R12-F	5’ CTGGTAGTCCACGCCGTAAAC 3’	This study
16S-C7R12-R	5’ CCAGGCGGTCAACTTAATGC 3’	This study
***hrp*A**		
HRPA-C7R12-F	5’ GGAAACCATCAAGAAGCAAACC 3’	This study
HRPA-C7R12-R	5’ GAGTCATCCTTGCCCGATTG 3’	This study

*All primers used in this study were synthesized by Eurogentec
